# A unique olfactory bulb microcircuit driven by neurons expressing the precursor to glucagon-like peptide 1

**DOI:** 10.1038/s41598-019-51880-9

**Published:** 2019-10-29

**Authors:** Nicolas Thiebaud, Fiona Gribble, Frank Reimann, Stefan Trapp, Debra Ann Fadool

**Affiliations:** 1 The Florida State University, Department of Biological Science, Program in Neuroscience, Tallahassee, USA; 2 Institute of Metabolic Science, University of Cambridge, Addenbrooke’s Hospital, Cambridge, UK; 3 Department of Neuroscience, Physiology and Pharmacology, University College London, London, UK; 4 The Florida State University, Institute of Molecular Biophysics, Tallahassee, USA; 5 Present Address: Division of Applied Regulatory Science, Center for Drug Evaluation and Research, U.S. Food & Drug Admnistration, Silver Spring, USA

**Keywords:** Olfactory bulb, Sensory processing

## Abstract

The presence of large numbers of local interneurons in the olfactory bulb has demonstrated an extensive local signaling process, yet the identification and purpose of olfactory microcircuits is poorly explored. Because the discrimination of odors in a complex environment is highly dependent on the tuning of information by local interneurons, we studied for the first time the role of preproglucagon (PPG) neurons in the granule cell layer of the olfactory bulb. Combining electrophysiological recordings and confocal microscopy, we discovered that the PPG neurons are a population of cells expressing the precursor of glucagon-like peptide 1 and are glutamatergic; able to modulate the firing pattern of the mitral cells (M/TCs). Optogenetic activation of PPG neurons resulted in a mixed excitation and inhibition that created a multiphasic response shaping the M/TCs firing pattern. This suggests that PPG neurons could drive neuromodulation of the olfactory output and change the synaptic map regulating olfactory coding.

## Introduction

The architecture of neuronal circuitry drives the capacity for information processing in the brain and the resultant output of an organism’s response. Amongst the wide array of microcircuits in the olfactory bulb, lie the GABAergic interneurons that shape olfactory information by controlling the contrast and gain of the signal in mitral/tufted cells (M/TCs). M/TCs, in turn, relay olfactory information to higher processing areas in the central nervous system^1^. A variety of cell types in the granule cell layer (GCL) had been identified as early as the beginning of the 20^th^ century^2,3^, however, little is still known concerning subclass-specific functions to date. As a consequence, the complexity of the network that encodes olfactory sensory information is often presented as highly oversimplified.

Previously we reported a subclass of deep short-axon cells (dSACs) located in the GCL, which we speculated to be responsible for the secretion of the traditional gut peptide, glucagon-like peptide 1 (GLP-1). We subsequently called these cells preproglucagon (PPG) neurons^4^. The exact function of PPG neurons in the olfactory system, especially their connection with other principal neurons and interneurons, is not understood. Recent publications linking olfaction and metabolic homeostasis have afforded an elevated interest in the role of olfactory neuromodulators and circuits in the control of body weight and obesity^4–7^. The presence of large numbers of local interneurons in the olfactory bulb has demonstrated an extensive local signaling process, not unlike that of the plexiform layers of the retina in the visual system, yet the identification and purpose of some olfactory microcircuits remains to be explored.

We designed a refined peroxidase labelling strategy to reveal that the PPG neurons have stellate dendrites covered with many spines, which are characteristic of dSACs^2,3,8–10^. The PPG neuron cell bodies were located in the inner part of the GCL with their axons projecting towards the internal plexiform layer and the mitral cell layer similar to the EPL-dSACs described by Eyre and collaborators^9,10^. Our data suggested that PPG neurons could synapse directly with the M/TCs to regulate their activity.

Herein, we investigated the PPG neuron to M/TCs unique microcircuit due to its potential important role in controlling olfactory output. We employed a new transgenic mouse model expressing CRE-recombinase under the control of the glucagon promoter to determine the expression of PPG, the precursor of glucagon and GLP-1. In acute slice preparations of the OB, where we could elicit light-activation of PPG neurons expressing channelrhodopsin-2 (ChR2), we determined the first biophysical properties for PPG neurons and recorded the consequences of their stimulation with postsynaptic M/TCs responses. Interestingly, the activation of PPG neurons evoked both inhibitory (IPSCs) and excitatory (EPSCs) postsynaptic currents in M/TCs that were abolished after application of glutamatergic inhibitors. Recording under current-clamp conditions, a biphasic inhibition-excitation control of action potential firing in M/TCs was found as a result of light-activation of the PPG neurons. Taken together, these results demonstrate that PPG neurons constitute a unique population of glutamatergic neurons within the GCL that form a local microcircuit controlling M/TC activity. Moreover, subsequent confocal imaging microscopy confirmed the expression of the vesicular glutamate transporter in PPG neurons.

Our study demonstrates a unique multiphasic control of M/TCs output involving mono- and di-synaptic connections and, for the first time, discovers a unique population of glutamatergic interneurons that were thought to be exclusively GABAergic. Such an unusual multiphasic modulation has important repercussions to play a key neuromodulatory role in regulating olfactory coding.

## Results

### Dual phenotype of PPG neurons

**Figure 1:**
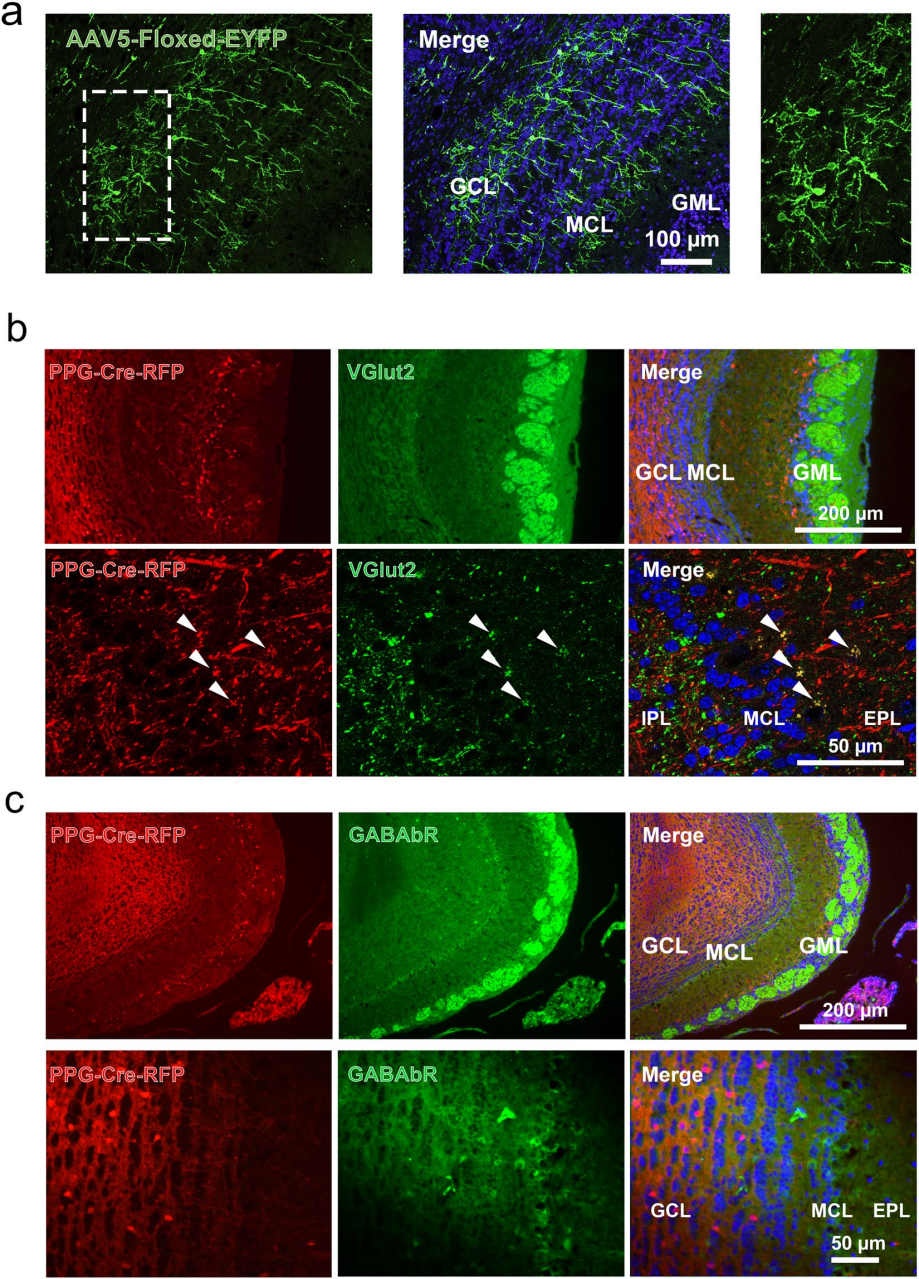
Preproglucagon (PPG) neurons express glutamatergic markers in the granule cell layer (GCL) of the olfactory bulb. (**a**) Left, representative photomicrograph of the GCL and mitral cell layer (MCL) in PPG-Cre-RFP transgenic mice transfected with AAV5 expressing ChR2-YFP conditional transgene (AAV5-Floxed-EYFP). Middle, same image (Merge) with DAPI nuclear stain. Right, enlarged magnification, inset delineated by the dashed box on left. (**b**) Representative photomicrographs of the glutamate transporter vGLUT2 visualized by immunocytochemistry in the external plexiform layer (EPL) of PPG-Cre-RFP transgenic mice. Arrow heads = co-expression of RFP (red channel; left) and vGLUT2 (green channel; middle) in synaptic terminals of PPG neurons in the EPL (merge; right).

Detection of preproglucagon (PPG) neurons expressing a red fluorescent protein (RFP) was achieved by crossing Rosa26-tandem-dimer red fluorescent protein (tdRFP) reporter mice^11^ with mice expressing Cre recombinase under the control of the glucagon promoter^12^. These mice will be referred to as PPG-Cre-RFP mice for simplicity. Morphological analysis demonstrated that the PPG neuron cell bodies were larger than granule cells (10.2 ± 0.2 μm, n = 73 from 3 mice) and were predominantly concentrated in the inner part of the granule cell layer (GCL) with axons projecting towards the mitral cell layer (MCL) and the internal plexiform layer (IPL) (Figs 1a, 3a,b). These findings were consistent with our previous study^4^. Granule cells are typically smaller than PPG neurons (range 6–9 μm) and mitral cells are significantly larger (range 25–35 μm)^3,8,13,14^. A manual counting of the total number of PPG neurons in the GCL gave an estimate of 10,076 ± 4,216 neurons per olfactory bulb. In comparison, Benson *et al*., 1984 report that there are 38,355 ± 781 MCs per olfactory bulb^15^. Estimation of the total number of granule cells is somewhat problematic and may increase with aging. Because the heterogeneity of cells in the GCL, a comprehensive count has not been reported, however, it is estimated that there is a 50–100:1 ratio between granule:mitral cells (Greer, Yale University, personal communication). We were able to confirm that the PPG-Cre-RFP mice exhibited the same pattern of PPG neuron expression as PPG-YFP mice that express the YFP variant Venus^16^ under the control of the mouse PPG promotor (mGLU-124 line)^17^. When PPG-Cre-RFP mice were injected at age P21 with an AAV expression of humanized ChR2 with H134R mutation fused to EYFP (see Methods), the mice exhibited EYFP-positive neurons in the GCL with stellar dendrites and numerous dendritic knobs as previously described for mGLU-124 mice^4^ (Fig. 1a). In addition to PPG-neurons in the GCL, we noticed a second population of fluorescent neurons in the glomerular cell layer (GML) extending lateral projections across multiple glomeruli, suggesting that these neurons could be classical short axons cells. This second population was not further explored in our study.

**Figure 2:**
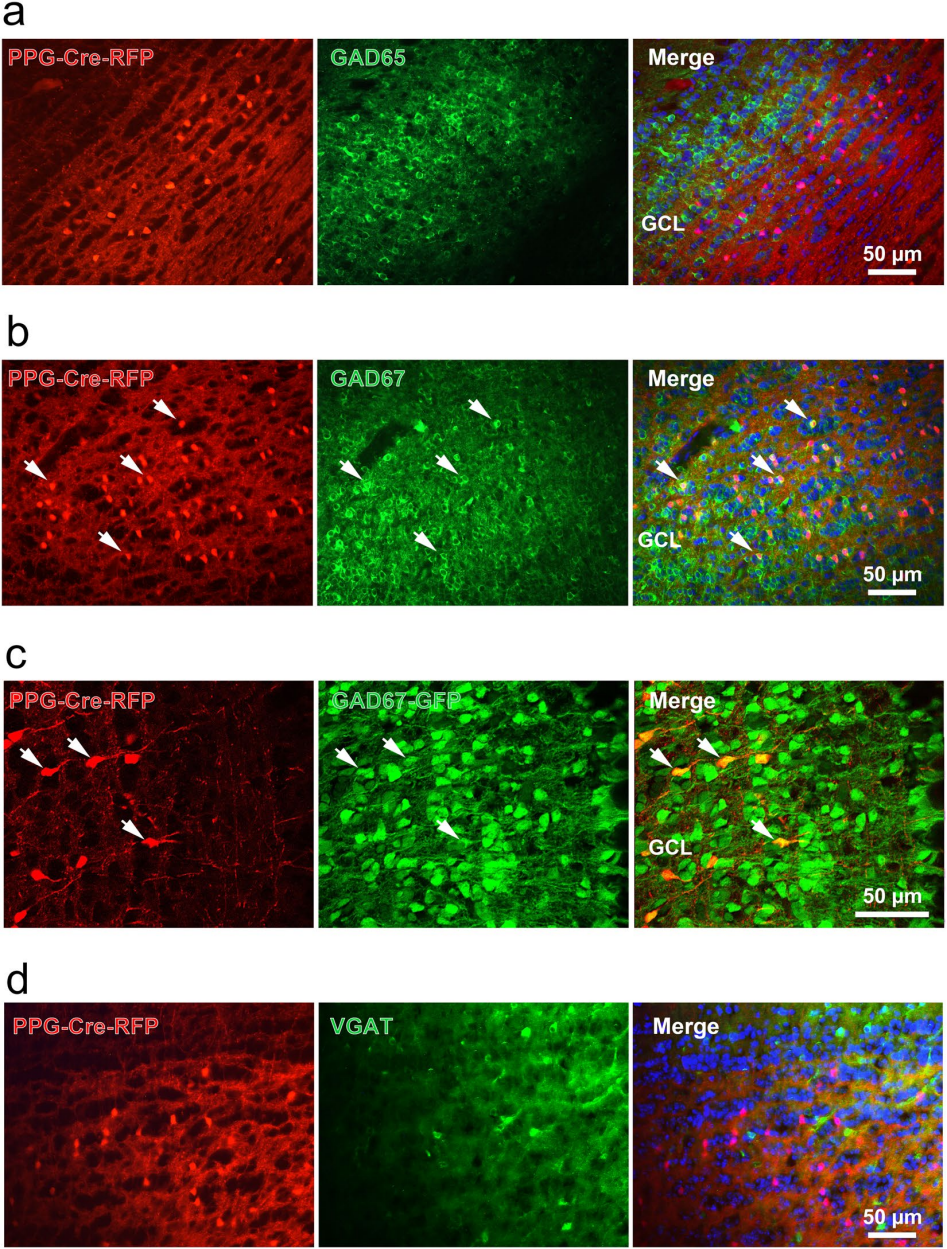
PPG neurons express glutamic acid decarboxylase 67 but not 65. (**a**) Representative photomicrograph of the GCL in PPG-Cre-RFP transgenic mice (red channel; left) whereby glutamic acid decarboxylases (GAD) GAD65 and (**b**) GAD67 are visualized using immunocytochemistry (green channel; middle). (**c**) Representative photomicrograph of the GCL in progeny resulting from a cross between PPG-Cre-RFP x GAD67-GFP mice. Arrow = PPG neurons that co-express RFP and GAD67. PPG neurons largely did not express GAD65 as evidenced by a lack of RFP and GAD65 co-labeled neurons. (**d**) Representative photomicrograph of the GCL in PPG-Cre-RFP transgenic mice (red channel; left) where the vesicular GABA transporter (VGAT) is visualized by immunohistochemistry (green channel; middle).

Because the PPG neurons residing in the GCL putatively appeared to be a type of dSAC, we used immunocytochemical approaches to examine the synaptic profile previously characterized for this cell type in rats^9,10^. Three-dimensional, 1-μm-thick Z-stack confocal images were acquired from PPG-Cre-RFP mice olfactory bulbs that were immunolabelled for the vesicular glutamate transporter 2, vGLUT2. As revealed in Fig. 1b, there was co-localization of the red fluorescence (tdRFP reporter) and green labelling of vGLUT2 in the axon synaptic terminals of PPG neurons in the internal part of the external plexiform layer (EPL) close to the MCL. Although difficult to represent on a single micrograph, co-localization of vGLUT2 and tdRFP was observed across the entire EPL of the olfactory bulb. Glutamate decarboxylase of isoforms 65 and 67 (GAD65/67) immunolabelling was then performed to further assess the specific phenotype of the PPG neurons. The red fluorescent signal attributed to the PPG neurons did not overlap with that of the green fluorescent labelling of the GAD65 isoform (0/100 neurons from 2 mice; Fig. 2a), but showed 97% co-labelling with that of the GAD67 isoform (156/160 neurons from 2 mice; Fig. 2b). To confirm the expression of GAD67 in PPG neurons, we crossed the PPG-Cre-RFP mice to heterozygous Swiss Webster GAD67-GFP knock-in mice and examined the co-localization of GFP with td-RFP in the olfactory bulbs of the resultant progeny. In the mice expressing all three transgenes, we found 100% of the PPG neurons in the GCL co-expressed GFP (152/152 neurons from 4 mice; Fig. 2c); demonstrating parallel evidence for the immunochemical localization findings. Finally, and quite unexpectedly, we found that the expression of the GABA transporter vGAT was not found in PPG-neurons, suggesting the absence of GABA in these neurons (Fig. 2d). In fact, immunolabeling for a number of classic proteins found in rat models of dSACs were absent in the PPG-Cre-RFP mice including potassium channel, Kv2.1, muscarinic acetylcholine receptor (mAChR), metabotropic glutamate receptor mGluR1α, and the GABAa receptor. These collective data suggested a different synaptic phenotype than traditional dSACs, which lead us to explore a correlative biophysical function.

### Optogenetic control of PPG neurons

**Figure 3:**
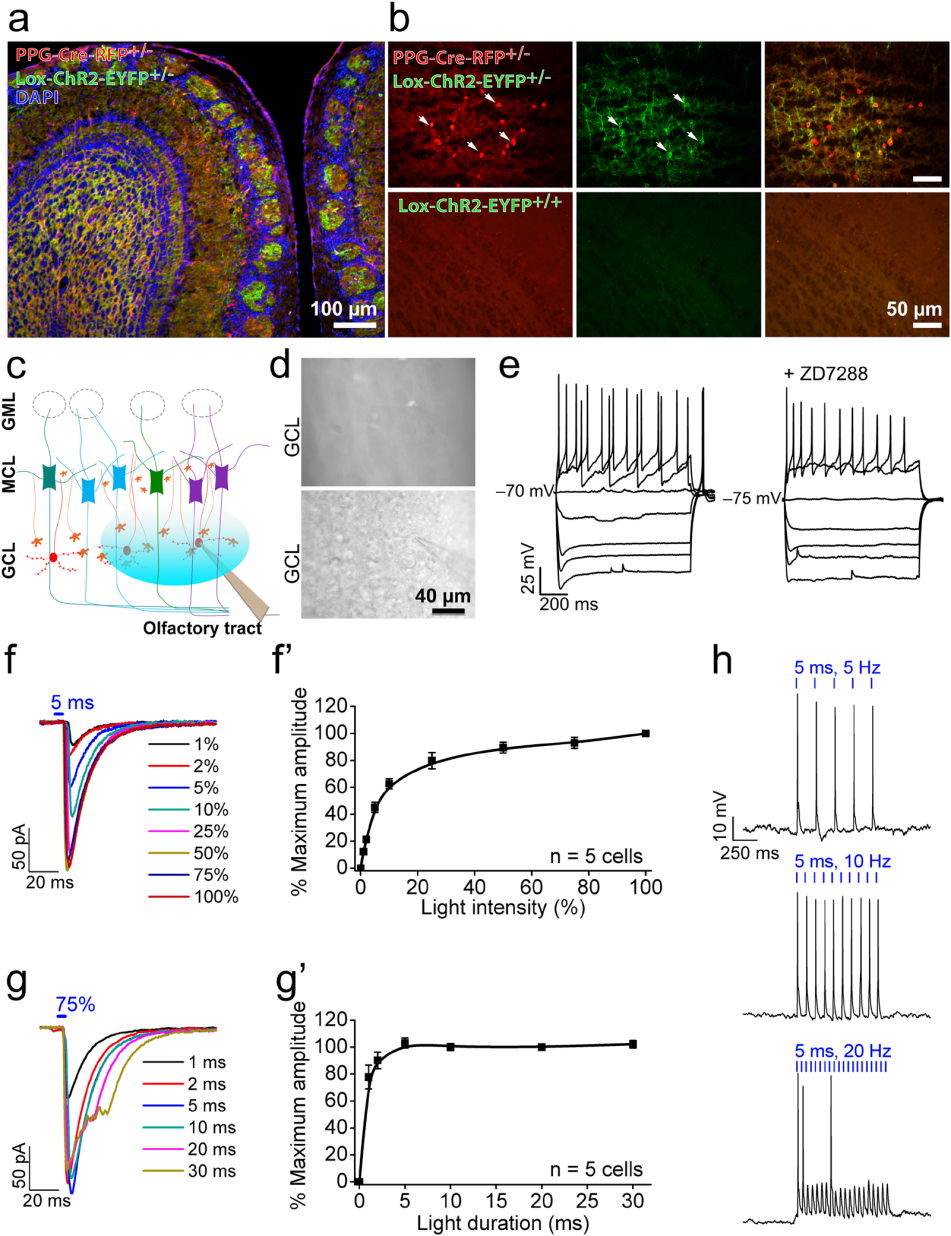
Conditional expression and optical activation of ChR2 in PPG neurons. (**a**) Representative photomicrograph acquired at low magnification in GLU-Cre12 x Rosa26tdRFP x ChR(H134R)-EYFP generated progeny (PPG-Cre-RFP^+/−^ Lox-ChR2-EYFP^+/−^). Note the robust co-expression of the red fluorescent protein in the PPG neurons (red channel) and that of the channelrhodopsin-2 (ChR2) (green channel) within the GCL, EPL, and projections into the glomeruli (**b**) (top) Higher magnification image of the GCL with PPG neurons (arrows; left) expressing the ChR2-eYFP on the membrane and dendritic arbors (middle). Merged overlay (right). (bottom) Floxed-ChR2 mice lack ChR2 expression when they are not crossed to PPG-Cre mice. (**c**) Schematic representation of the olfactory bulb and electrode placement for patch-clamp recordings from PPG neurons (red). The blue ellipse represents the zone in the GCL illuminated during the optogenetic light stimulation at 470 nm. orange = granule cells (GCs), green, blue, and purple = mitral cells projecting to independent glomeruli, GCL = granule cell layer, MCL = mitral cell layer, GML = glomerular cell layer. (**d**) Photomicrograph of a PPG neuron in the GCL contained in a 300 µm slice of olfactory bulb. (top) PPG neurons were identified using red fluorescence. (bottom) Merged overlay with bright-field. (**e**) Representative current-clamp recording of a PPG neuron in response to 25 pA current injections from –100 to 50 pA. Bath application of ZD7288 (+ZD7288; right). (**f**–**g**) Representative ChR2 currents in PPG neurons recorded in the presence of 1 µM TTX while variating light stimulation intensity (**f**,**f’**) or duration (**g**,**g’**). V_*hold*_ = –70 mV. The maximum current amplitudes are plotted in (**f’**) and (**g**’), respectively (n = 5 cells). (**h**) Current-clamp recordings showing action potential firing in PPG neurons after stimulation with 5, 10 and 20 Hz trains of light pulses (5 ms, 75% intensity).

To study the downstream synaptic consequences of activating PPG neurons within the olfactory bulb network, PPG-Cre-RFP mice were bred with Ai32 mice to heterozygosity. The Ai32 mice expressed an improved channelrhodopsin-2/EYFP fusion protein Cre recombinase-dependently in the PPG-Cre-RFP line^18^. As shown in Fig 3a,b, ChR2-EYFP was strongly expressed in the majority of PPG neurons in the dendritic arbors and the plasma membrane (Fig. 3b, upper panel). Ai32 mice that were not crossed with PPG-Cre-RFP mice did not exhibit any EYFP fluorescent signal, demonstrating the absence of ChR2 expression leakage (Fig. 3b, bottom panel). Because the PPG-cre-RFP x ChR2-EYFP progeny might label neurons outside the olfactory bulb that could centrifugally project back to the M/TCs to confound our interpretations, we histologically examined higher projections to confirm lack of yellow fluorescent signaling (data not shown). We found that EYFP-positive neurons in the GCL had dendrites extending across multiple glomeruli suggesting they were dSACs. The EYFP-positive neurons were mainly found in the GCL as previously reported^19^ although some were also found in the GML. Because light stimulation of the GML did not elicit any electrophysiological activity of MCs, we focused our optogenetic stimulation and further study exclusively on PPG neurons in the GCL.

To confirm the expression and define the activity of light-activated ChR2, we patch-clamped PPG neurons in the whole-cell configuration as guided by the expression of RFP (Fig. 3c,d). PPG neurons could be differentiated from granule cells (see Table 1) by their discrete biophysical properties that included - high input resistance (850 ± 150 MΩ, n = 15), greater cell capacitance (15.7 ± 0.6 pF), specific resting potential (−75.1 ± 1.6 mV), and the absence of spontaneous firing activity in current-clamp. No spontaneous action potentials were observed in the cell-attached configuration that keeps the intracellular composition unchanged. In response to the injection of current steps ranging from −100 to + 50 pA, we noticed the presence of a sag and a rebound spike, which usually depicts the presence of a hyperpolarization-activated current (I_h_) (Fig. 3e). This hypothesis was verified by adding a selective blocker of I_h_ current, ZD7288, to the bath solution, which resulted in the suppression of the sag and a reduction in the rebound spiking.

**Table 1:** Intrinsic membrane properties and light-elicited currents in olfactory bulb neurons.

Cell type	PPG-neuron	Granule Cell	M/T Cell			
***Membrane properties***						
Input resistance (MΩ) (n)	850 ± 154 (15)	748 ± 330 (7)	142 ± 5 (195)			
Capacitance (pF)	15.71 ± 0.63	10.35 ± 1.52	90.25 ± 1.71			
Resting potential (mV)	–75.07 ± 1.63	N/A	–64.39 ± 0.59			
Spontaneous action potentials	None	Yes	Variable			
***Light-induced current properties*** ^#^						
**Current type**	**ChR2**	**EPSC**	**IPSC**	**EPSC**	**IPSC**	**EPSC**
V_hold_	−70 mV + 1 µM TTX	−70 mV	+ 0 mV	−70 mV	+ 0 mV	−70 mV
Intracellular solution (n)	KGluc (8)	CsMeSO_3_ (6)	CsMeSO_3_ (9)	CsMeSO_3_ (9)	KGluc (15)	KGluc (15)
Latency (ms)	0.13 ± 0.04	12.50 ± 1.60	11.17 ± 0.58	6.69 ± 1.09***	8.59 ± 0.74	3.18 ± 0.29***
Amplitude (pA)	118.46 ± 29.24	78.04 ± 5.78	245.00 ± 44.64	87.38 ± 16.46***	38.33 ± 5.28	17.75 ± 2.36***
Rise time (ms)	2.89 ± 0.91	2.61 ± 0.65	6.73 ± 0.66	9.36 ± 3.74	5.23 ± 1.34	12.74 ± 2.21***
Rise slope (pA/ms)	52.16 ± 23.25	25.75 ± 4.31	25.37 ± 6.08	10.86 ± 3.28***	7.41 ± 1.79	1.24 ± 0.34***
Decay time (ms)	18.27 ± 1.36	15.31 ± 12.32	39.38 ± 3.84	102.62 ± 79.48*	15.07 ± 2.41	51.59 ± 6.49***
Decay slope (pA/ms)	3.46 ± 0.87	5.82 ± 1.48	3.69 ± 0.71	2.19 ± 0.64**	1.85 ± 0.53	0.23 ± 0.05***
½ width (ms)	12.62 ± 0.37	5.27 ± 2.63	31.83 ± 1.26	30.28 ± 8.85	15.34 ± 2.01	39.82 ± 5.76***
Area (pA.ms)	2710 ± 909	1186 ± 247	10200 ± 2093	3427 ± 1125***	763 ± 143	1045 ± 206***
Synaptic blockers sensitivity	None	AP_V _ + NBQX	GBZ and AP_V _ + NBQX	AP_V _ + NBQX	GBZ and AP_V_ + NBQX	AP_V _ + NBQX

Each value was calculated from the average of 5–10 consecutive traces and represents the mean ± SD.

^#^Recorded after a 5 ms light-stimulus (470 nm), 75% of total intensity. Responses >20 ms were not considered light-induced events and were excluded from analyses.

IPSC vs EPSC *p ≤ 0.05, **p ≤ 0.001, ***p ≤ 0.0001. Student's *t*-test.

Next, we investigated the response of PPG neurons to 473 nm light activation of the ChR2, and determined the minimum duration and intensity of light exposure in order to minimize phototoxicity. Voltage-clamp recordings were performed in the presence of the Na^+^ channel blocker, tetrodotoxin (TTX, 1 µM), to isolate ChR2 currents and avoid action-currents generated from depolarization of the neuron. The latency measured from the beginning of the light stimulation (<1 ms) and the neuron’s insensitivity to TTX and synaptic blockers confirmed that the currents recorded were intrinsic to the PPG neurons and elicited by ChR2 activation (Table 1). We found that a 5 ms stimulus (Fig. 3f,f’) with the light intensity set to 75% (Fig. 3g,g’) was sufficient to obtain a maximum current amplitude (118.5 ± 29.2 pA, Table 1) in most of the PPG neurons. In current-clamp mode, light exposure using these parameters evoked consistent action potentials at 5 and 10 Hz frequencies, although signs of spike adaptation were observed at higher frequencies where suprathreshold depolarizations due to ChR2 currents were observed but failed to elicit action potentials (20 Hz) (Fig. 3h).

### PPG neurons to M/TCs connection

**Figure 4:**
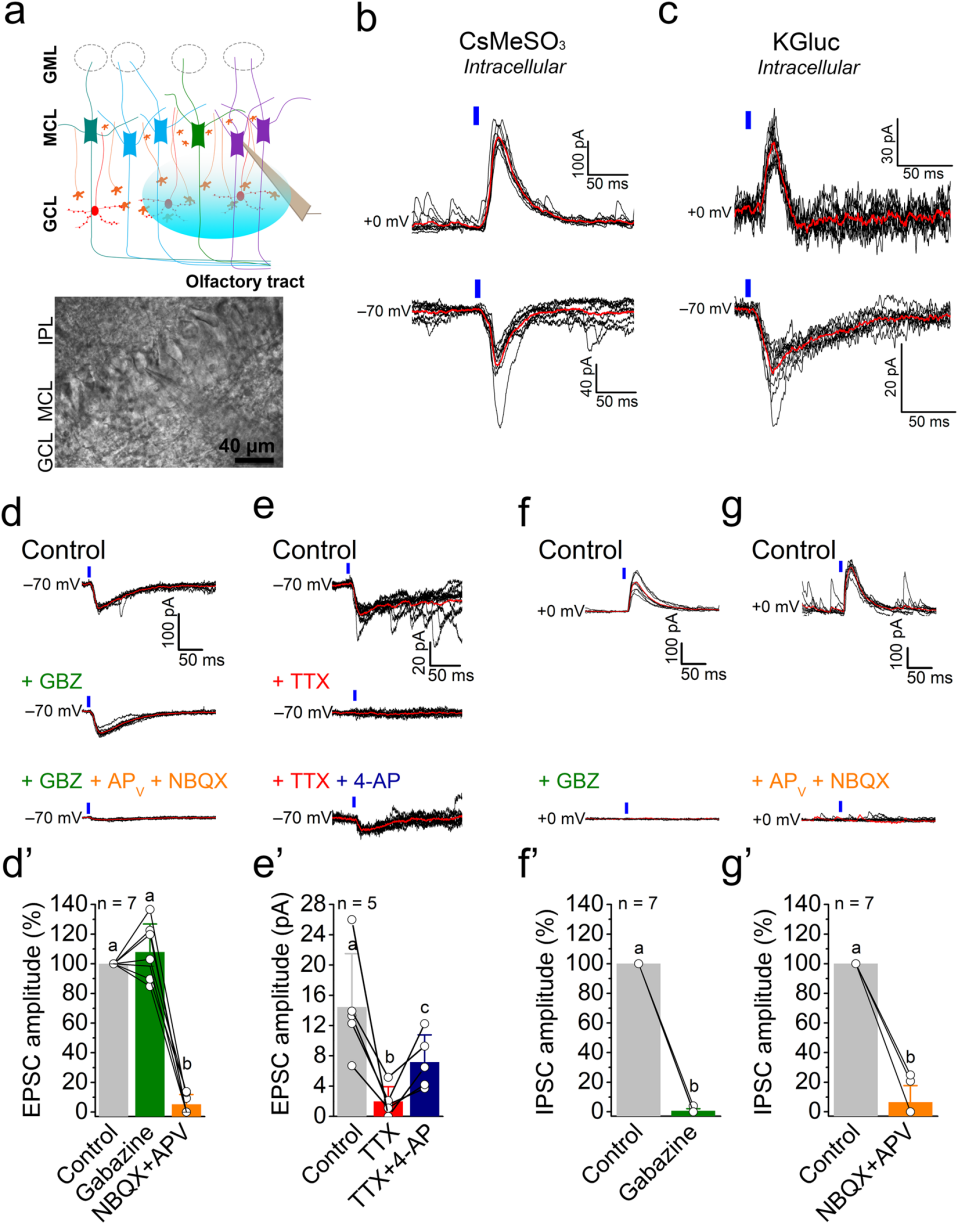
Light-stimulation of PPG neurons in the GCL induces inhibitory- and excitatory-postsynaptic currents in mitral cells. (**a**) (top) Schematic representation as in Fig. 2 with electrode placement on the M/TCs and optical stimulation of the GCL (blue ellipse). (bottom) photomicrograph of electrode placement on a mitral cell in the GCL of a 300 µm slice of OB. (**b**) Light-induced inhibitory- (IPSC, V_*hold*_ =  + 0 mV) and excitatory- (EPSC, V_*hold*_ = –70 mV) postsynaptic currents recorded in response to light stimulation (blue vertical bar) of PPG neurons using cesium-methane-sulfonate (CsMeSO_3_) internal solution or, (**c**) potassium-gluconate (KGluc) internal solution. (**d**,**d’**) (top) Light-induced EPSCs in a single M/TC under control ACSF conditions (Control), (middle) following bath application of the GABA_B_ receptor antagonist gabazine (10 µM GBZ), (bottom) and then with the addition of the glutamate blockers (100 µM AP_V_, and 20 µM NBQX), (**e**,**e’**) (top) Light-evoked EPSCs in a single M/TC under control ACSF conditions (Control), (middle) following bath application of 1 µM TTX, (bottom) and then with the addition of 1 mM 4-AP. (**f**,**f’**) (top) Light-induced IPSCs in a single M/TC under control ACSF conditions (Control), (bottom) and following bath application of GBZ, (**f**,**f’**), (top) light-induced IPSCs in a single M/TC under control ACSF conditions (Control), (bottom), and following bath application of AP_V_ and NBQX. (**b**–**g**) Vertical blue bars (light stimulation) = 5 ms, 75% intensity. Red traces = mean of 8–10 recordings. (**d’**–**g’**) Values noted with the same letter are not significantly different. Non-parametric Friedman test followed with a Dunn’s multiple comparison test (**d’**,**e’**) or Wilcoxon matched-pairs signed rank test (**f’**,**g’**).

The connection between PPG neurons and M/TCs was investigated by placing the recording electrode on the M/TCs, while light-stimulating the PPG neurons in the GCL using the Mightex illuminator that allows a precise spatial and temporal light illumination on the brain slice (Fig. 4a). Because M/TCs post-synaptic currents can differ depending on the internal solution^20^, we compared cesium-methane sulfonate (CsMeSO_3_)- vs. potassium gluconate (KGluc)-based internal solutions to record light-elicited, post-synaptic currents generated by PPG neuron activation. The control of holding potential (V_hold_) allowed us to isolate either inhibitory- or excitatory-postsynaptic currents (IPSC, V_hold_ =  + 0 mV; EPSC, V_hold_ = −70 mV) on the same cell (Fig. 4b,c). Interestingly, we found that a single light pulse on the PPG neurons (5 ms, 75%) was sufficient to elicit both EPSCs and IPSCs in M/TCs (Fig. 4b). As expected, currents recorded using CsMeSO_3_ had a larger amplitude than those using potassium-gluconate based solution (KGluc) for both IPSCs and EPSCs (Table 1), due to the inhibition of K^+^ currents, which allowed the conservation of currents elicited distantly from the soma. Postsynaptic events had notably longer onset latencies and time constants in CsMeSO3 than in KGluc based internal solutions. The light-evoked IPSCs had longer latencies than that of the EPSCs for both internal solutions tested (IPSCs = 11.1 ± 0.6 ms vs EPSCs = 6.7 ± 1.1 ms for CsMeSO_3_ based internal solution, and IPSCs = 8.6 ± 0.7 vs EPSCs = 3.2 ± 0.3 ms for KGluc solution; Table 1). Light-induced EPSCs had longer decay constants (decay time = 102.6 ± 79.5 ms, decay slope = 2.2 ± 0.6 pA/ms in CsMeSO_3_ internal solution; Table 1) when compared to IPSCs (decay time = 39.4 ± 3.8 ms, decay slope = 3.7 ± 0.7 pA/ms in CsMeSO_3_ internal solution; Table 1) revealing a longer excitation than inhibition of the M/TCs. It is noteworthy that some of EPSCs recorded from M/TCs present a very slow decay time >1 s. In addition, latency jitter, i.e. the SD of the latency of the EPSCs recorded were shorter (0.182 ± 0.025 µs) than for IPSCs (1.422 ± 0.926 µs).

We then investigated the nature of the synaptic connection between PPG neurons and M/TCs by applying synaptic blockers to the bath solution (Fig. 4d–f’). As we anticipated, EPSCs were not affected by the GABA_A_ antagonist, gabazine (GBZ, 10 µM), but were almost abolished after AMPA/Kainate and NMDA glutamate receptor antagonists, NBQX (10 µM) and AP_V_ (50 µM), suggesting a glutamatergic origin (Fig. 4d,d’). Surprisingly and contrarily, IPSCs were totally abolished both following GBZ application (100%, n = 7 cells; Fig. 4f,f’) and also after application of glutamate blockers (94%, n = 7 cells; Fig. 4g,g’), suggesting that the inhibitory input from PPG neurons on the M/TCs had a multi-synaptic origin. This observation was in accordance with the greater latency and jitter of IPSCs compared to that of the EPSCs (Table 1). To determine if the glutamatergic EPSCs were mono- or multi-synaptic in origin, we used a previously-described technique^21^ to depolarize ChR2-positive presynaptic termini directly by blocking action potentials using TTX (1 µM) and blocking potassium channels using 4-aminopyridine (4-AP, 100 μM). TTX prevents recurrent activity whereas 4-AP is used to allow more efficient depolarization of axonal and presynaptic membranes, which thereby allows the release of presynaptic vesicles just by the action of ChR2 currents. The partial recovery of light-evoked EPSCs in the absence of any action potentials, and after application of 4-AP (Fig. 4e,e’), demonstrated that the excitatory input of PPG neurons on the M/TCs was monosynaptic.

### PPG neuron to GC connection

**Figure 5:**
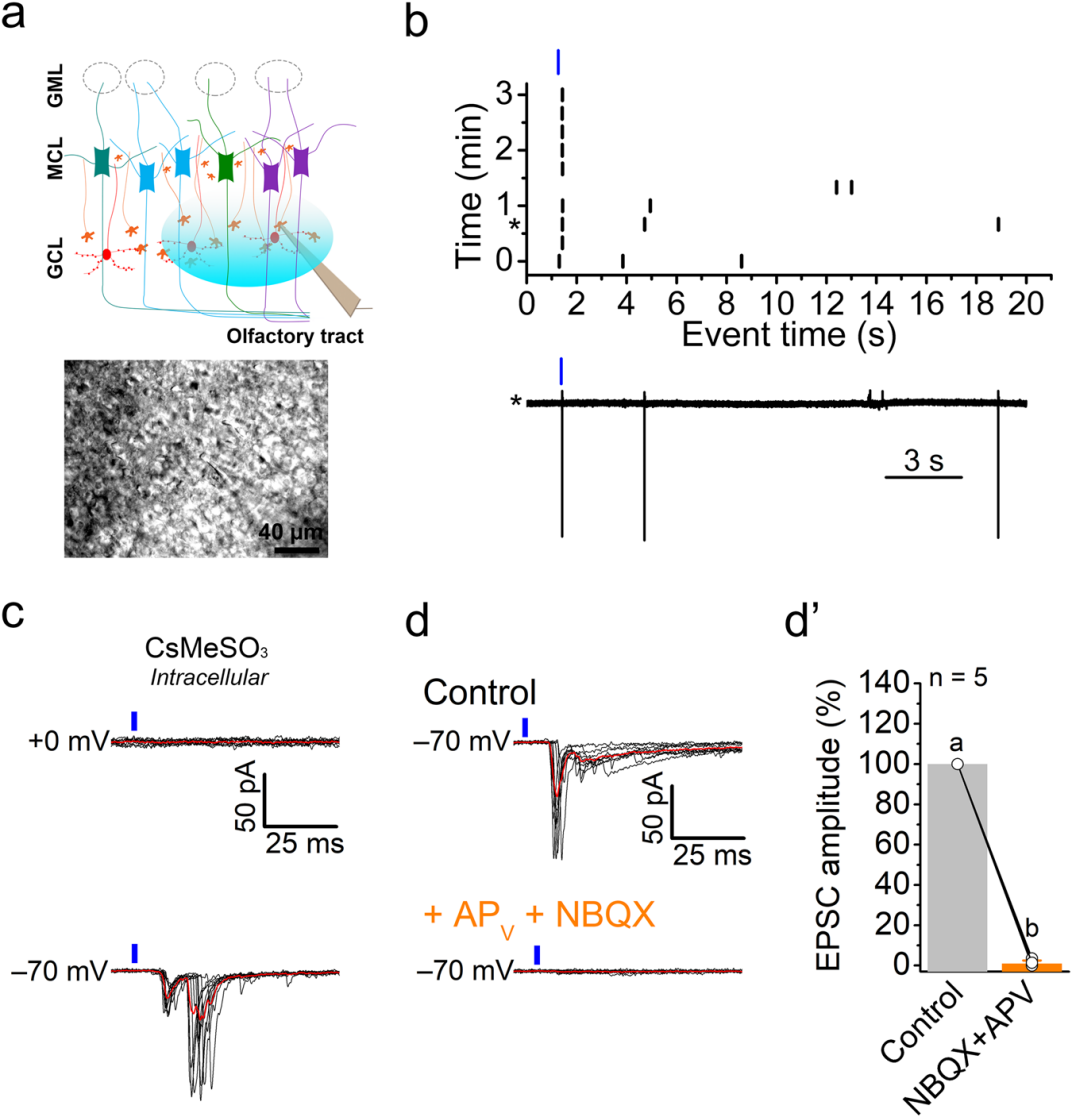
Light-stimulation of PPG neurons in the GCL induces excitatory-postsynaptic currents in granule cells. (**a**) (top) Schematic representation as in Fig. 2 with electrode placement on the GCs, and optical stimulation of the GCL (blue ellipse). (bottom) Photomicrograph of electrode placement on a granule cell in the GCL. (**b**) Raster plot representing cell-attached recordings from a granule cell and (*) a representative recording. (**c**) (top) rGanule cells do not receive IPSCs (V_*hold*_ = + 0 mV) after light stimulation of PPG neurons, but (bottom) receive EPSCs (V_*hold*_ = −70 mV). (**d**,**d’**) (top) Light-evoked EPSCs recorded in a single GC under control ACSF condition (Control) and (bottom) following bath application of AP_V_ and NBQX. Statistical analyses and notations as in Fig. 3f’,g’.

GCs provide GABAergic inhibitory input to the M/TCs through a rapid feedforward inhibition involving dendrodendritic M/TC to GC glutamatergic stimulation, followed by a release of GABA from the GCs to the M/TCs^22^. Consequently, we investigated if the multi-synaptic IPSCs previously recorded on the M/TCs following light activation of the PPG neurons could originate from the activation of granule cells. Recordings were thus performed directly on GCs following light-stimulation of PPG neurons (Fig. 5a). In a cell-attached configuration that allows the recording of small cells without interfering with their intracellular composition, we found that a single pulse light stimulation of PPG neurons generated action currents in the GCs (Fig. 5b) indicating that this was a stimulating synaptic input to the GCs (n = 7). Recordings made in the whole-cell configuration showed that single light-pulses failed to generate IPSCs but were able to generate light evoked EPSCs in some GCs (6/13) (responses with a latency larger than 20 ms were considered spontaneous events and not light-evoked and were omitted from the analysis (outlier’s test), 78.0 ± 5.7 pA, n = 6; Fig. 5c, Table 1). Similarly to the M/TCs, EPSCs were blocked after bath application of the glutamatergic blockers AP_V_ and NBQX (Fig. 5d,d’), however, the latency of the generated EPSCs was significantly longer than those recorded in M/TCs (12.5 ± 1.6 ms in GCs vs 6.7 ± 1.1 ms in M/TCs, Table 1) and they presented an asynchronous pattern (Fig. 5c,d). This asynchronous pattern of light-evoked EPSP suggested that the activation of the GCs was the result of PPG to M/TC monosynaptic activation, which in turn triggered a M/TC-to-GC glutamate release. The latency of light-evoked EPSCs in GCs was not significantly different from the latency of the IPSCs recorded in the MCs (12.5 ± 1.6 ms vs 11.17 ± 0.58 ms, Table 1) consistent with the inhibitory response observed at the M/TC level being the result of feedforward inhibition at the MC/GC synapse as discussed below.

### PPG neurons elicit complex response in M/TCs

**Figure 6:**
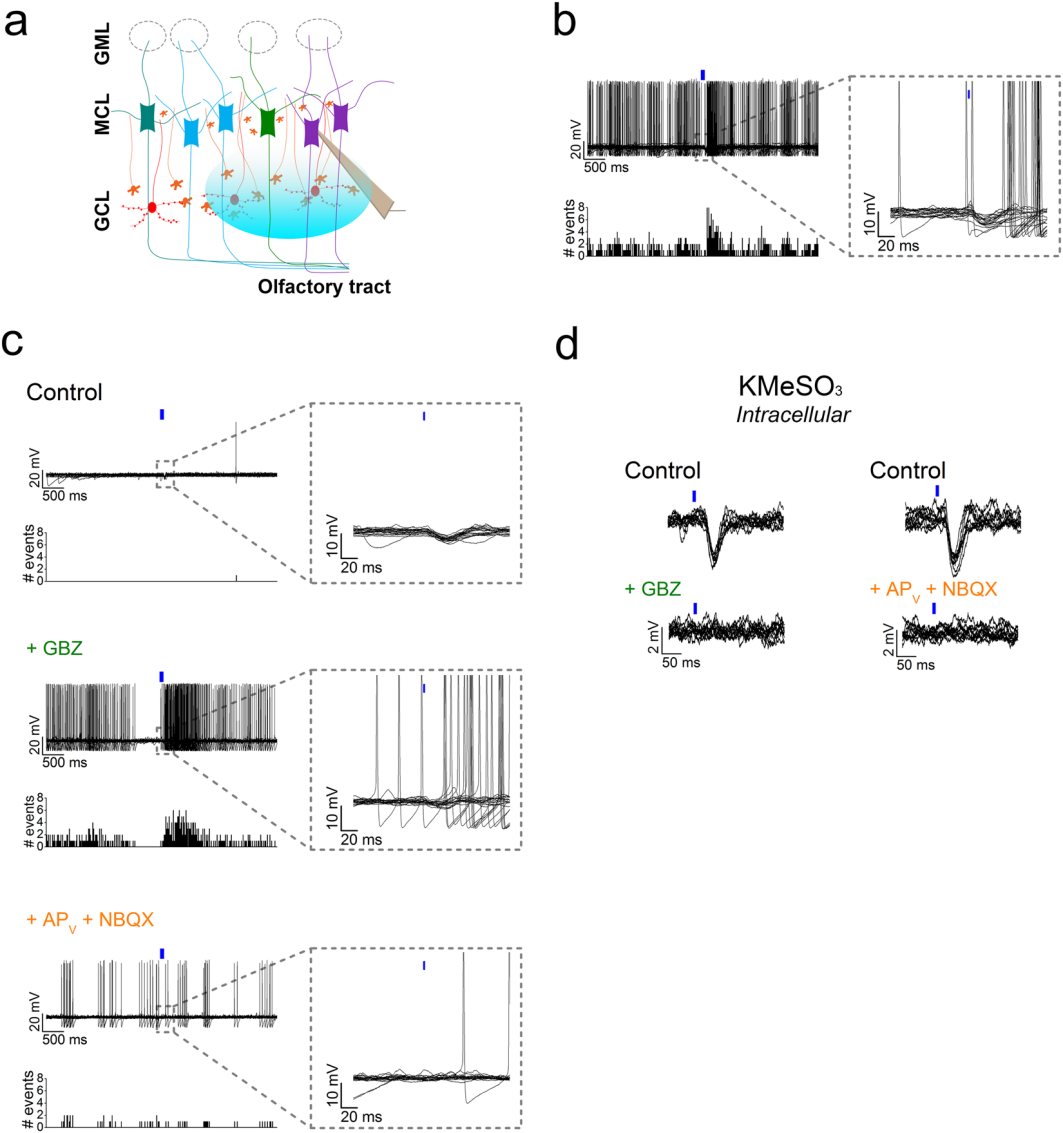
Light-stimulation of PPG neurons induces a biphasic inhibition-excitation response in mitral cells. (**a**) Schematic representation as in Fig. 2 with electrode placement on the M/TC, and optical stimulation of the GCL (blue ellipse). (**b**) (top, left) Twenty current-clamp traces recorded from a M/TC showing a biphasic inhibition-excitation response after light-stimulation (tick) of PPG neurons, (dashed inset) altered time scale resolution of the light-stimulation event (right). (bottom, left) Peristimulus time histogram (PSTH) of all events (20 ms bins). (**c**) Different example of a silent M/TC where light-stimulation induces an inhibitory post-synaptic potential (IPSP) only. (top) Recordings of a single M/TC under ACSF conditions (Control), (middle) following bath application of GBZ, and (bottom) and then following bath application of AP_V_, and NBQX. (**d**) Current-clamp recordings from a M/TC where light-induced IPSPs are observed using potassium-methane-sulfonate (KMeSO_3_) as the internal solution. (top) Using ACSF conditions (Control) and following (bottom left) bath application of GBZ or (bottom right) AP_V_ + NBQX.

How does the dual inhibitory/excitatory input of PPG neurons influence M/TCs? To answer this question, we performed current-clamp recordings on M/TCs while stimulating the PPG neurons (Fig. 6a). For this experiment, M/TCs were maintained at their resting potential (~ −65 mV; Table 1). Overall, 65% of M/TCs were found to respond to a single 5 ms, 75% light stimulation of the PPG neurons. Whether the non-responding cells did not receive inputs from PPG neurons, or the input stimulation was too weak to record any change in the firing frequency, or if the connections were lost during the olfactory bulb slice preparation remained unresolved. M/TCs can either show spontaneous bursting firing, or they are silent or have low basal activity^23,24^. In M/TCs that exhibit spontaneous bursting, a single light pulse elicited a triphasic response, consisting of a slight depolarization, followed by a hyperpolarization, and a subsequent rebound excitation (Fig. 6b). This response was directly in agreement with our voltage-clamp observations in Fig. 4, where on average EPSCs displayed shorter latency and slower decay times compared to IPSCs, meaning a longer excitatory input. In other cases, M/TCs had much lower spontaneous firing activity as presented in Fig. 6c. These cells received stronger inhibitory inputs from inhibitory interneurons as depicted by numerous spontaneous inhibitory post synaptic potentials (sIPSPs). M/TCs with a low basal activity responded to the light-activation of PPG neurons hyperpolarization only (Fig. 6c, top trace). Interestingly, addition of the GABA_A_ antagonist GBZ resulted in increased spontaneous firing, due to the inhibition of inhibitory input from interneurons, a suppression of the light-evoked hyperpolarization, followed by an increase in the excitation (Fig. 6c, middle trace). Consistent with our voltage-clamp observations in Fig. 4, when we applied glutamatergic receptor blockers, the light response was completely abolished (Fig. 6c, lower trace).

Because the resting potential of MCs is close to the reversal potential for GABA_A_-receptors, inhibitory post synaptic potentials can be masked when recording in the current-clamp mode. To overcome this, we replaced the conventional KGluc internal solution with potassium methane sulfonate (KMeSO_3_) that displaced the chloride reversal potential to −110 mV, lower than the resting potential of M/TCs. KMeSO_3_ increased the light-induced hyperpolarization or inhibitory postsynaptic potential (IPSP) but did not reveal any excitatory inputs on M/TCs (Fig. 6d). Similarly to IPSCs, light-induced IPSPs were totally suppressed after bath application of either GBZ or AP_V_/NBQX (Fig. 6d).

## Discussion

The design principles and function of microcircuits are not yet well understood but serve as elementary processing units linking single neurons to global brain function and ultimately behavior^25^. The dSACs remain one of the most understudied cell types in the olfactory bulb neuronal network. Microscopic analyses have estimated a total number of around 13,500 dSACs in the rat olfactory bulb^9,10^ which, compared to the MC total population (50,000), suggests that the dSACs and the microcircuit they define is substantial with regards to olfactory bulb processing. Our study provides the first evidence that terminations of some GLP-1 producing neurons in the inner part of the granule cell layer (GCL) are capable of providing a fine tuning of M/TC output by activating both excitatory glutamatergic synapses and an inhibitory GABAergic response.

We show that PPG neurons represent a unique class of interneurons by mediating monosynaptic excitation on MCs. PPG neurons are a particular subclass of olfactory bulb interneurons of the GCL^4^; more precisely, their slightly larger soma compared to GCs, and their stellar dendrites with dendritic buttons suggest they are dSACs. Using a rigorous molecular and anatomical characterization of dSACs in the GCL of rats, Eyre and collaborators^9^ identified and classified three major dSAC subtypes based upon the projection of their axons, namely: granule cell layer (GCL)-, external plexiform layer (EPL)-, and glomerular layer (GL)-dSACs. A further refinement of neuronal classification within the glomerular layer (GL) was recently made by Tavakoli *et al*. (2018) utilizing morphological, physiological and multivariate analysis, with the similar purpose to provide better classificaiton for microcircuit studies^26^. Due to the axonal projections of dSACs to either the glomerular or external plexiform layer, one would propose a regulation of olfactory output by regulating the electrical output of M/TCs. Based on the classification established by Eyre and colleagues, the localization in the central part of the GCL and the projection of their axons to the external plexiform layer and mitral cell layer, PPG neurons can be described as EPL-dSACs^9,10^. Only few previous studies have characterized the role of dSACs and all those suggested an inhibitory GABAergic activity. Boyd *et al*. (2012) found that some dSACs, receiving excitatory projections from the piriform cortex, drive a direct feedforward inhibition of GCs^27^. Burton and colleagues (2017) extensively studied the interneuron features of GL-dSACs, another class of dSACs located in the internal plexiform layer that sends their projections to the GL^28^. GL-dSACs receive convergent external tufted cell (ETC) excitation, mediate the inhibition of tufted cell apical dendrites, and deliver selective output onto interneurons and principal tufted cells. These authors recorded excitation of periglomerular cells that they attributed to an excitation by GABA on depolarized Cl^-^ reversal potentials triggering GABA-induced GABA release^28^.

Some differences can be denoted between dSACs described by previous studies and the PPG neurons we described in this study. For example, dSACs characterized by Eyre and colleagues using electron microscopy indicated that dSAC terminals targeted GC dendrites but did not form direct synaptic contacts with M/TCs^9^. It must be noted that all previous studies have been performed in rats, thus the differences we observed in the present study such as the lack of expression of Kv2.1 or the muscarinic acetylcholine receptor 1α may be the result of differences between species. These data add further evidence that PPG neurons, which have a monosynaptic connection to M/TCs, are a different population of dSACs compared to those characterized in previous studies. It is possible that they did not sample this connection in their microscopic analysis. Other studies using paired-recordings have shown that dSACs generate spontaneous IPSCs onto GCs^29^. The fact that we did not record spontaneous firing in the PPG neurons, whether in whole-cell or cell-attached mode, further suggests that these neurons are electrophysiologically distinct from those dSAC previously reported^9,28,30^.

Interestingly, although PPG neurons exhibit a dual glutamatergic and GABAergic phenotype with the co-expression of both GAD67 and vGLUT2, we did not find evidence of a direct release of GABA from PPG neurons onto M/TCs or GCs. Such a duality is not unique in the OB, for instance in the glomerular layer, Tatti and collaborators identified a population of GAD67-positive neurons co-expressing the vesicular glutamate transporter 3 (vGLUT3), which displayed electrophysiological properties of glutamatergic neurons^31^. Alternatively spliced or truncated versions of the GAD enzyme that synthesize GABA have been reported during developmental stages, which have yet no known function^32^. Another evidence of a glutamatergic phenotype lays in the lack of expression of the vesicular GABA transporter (vGAT) in the PPG-neurons. Moreover, in other areas of the brain, as in the paraventricular (PVN) and dorsomedial (DMH) hypothalamic nuclei, PPG neurons have been shown to co-express vGLUT2 and GLP-1 at the level of their axon varicosities, suggesting that glutamate is a co-transmitter at synapses within the hypothalamus^33,34^. At the synaptic level, GLP-1R signaling has been shown to modulate AMPA/Kainate glutamate receptor activity by increasing mEPSC frequency and decreasing the paired-pulse ratio in medium spiny neurons of the nucleus accumbens and the ventral tegmental area^35,36^. While we did not observe a significant change in pair-pulse ratio recorded from MCs (data not shown); this is consistent with our previous study that showed that GLP-1 directly increases the evoked spiking activity in MCs through an inhibition of the potassium channel Kv1.3^37^. These collective findings suggest a paracrine role of GLP-1 in the modulation of the olfactory output.

In exploring the intrinsic membrane properties and light-evoked currents in the PPG-MC-GC microcircuit, we encountered some complexities in the kinetics of the responses. Our latency data show that light-activation of PPG neurons evokes glutamate-induced EPSCs in MCs that are significantly faster than the GABA-induced IPSCs in MCs (Table 1). While this would be consistent with a classical dendrodendritic feedforward release of GABA from GCs to MCs, we also noted that the EPSCs in GCs were not significantly different in latency from the IPSCs in MCs. Although one would anticipate the IPSCs in the MCs to have the greatest latency, because they are the last one in this chain of events, it may be difficult to accurately measure them. Feedforward inhibition occurs within the dendrodendritic microcircuit of the MC/GC synapses and we were not able to make direct recordings of local currents within the dendrodendritic connection. Rather whole-cell recordings at the soma of MCs and GCs, respectively, both of which are remote to the synapse, were used to record these events. Therefore, the recorded kinetics reflect when the EPSC and IPSC have travelled to the respective soma. This is dependent on the exact cell morphology and the GC EPSC might reach the GC soma later than the MC IPSC might reach the MC soma due to the different architecture of the cells, despite the EPSC at the dendrodendritic synapse happening before the MC IPSC. An additional factor in accurately resolving the kinetics of activation within the synapses of this microcircuit are the size differences between the MCs and the smaller GC and PPG neurons. Finally, we cannot exclude that fibers of other centrifugal pathways could provide further complexity and lead to generation of inhibitory responses. The observation that some M/TCs could be exclusively inhibited in response to light-evoked PPG activity suggest that further multisynaptic inhibitory routes from PPG to M/TCs in addition to the observed M/TC-GC dendrodendritic M/TC-inhibition, exist.

In mammals, the olfactory bulb is the first stage of olfactory processing and is thereby crucial to shape the olfactory output by M/TCs. Microcircuits involving interneurons in the olfactory bulb have been shown to increase the gain control, filter weak inputs, or temporally shape and synchronize M/TCs spiking patterns, resulting in a reduction of the signal-to-noise ratio of odor representation in complex environments^38–40^. Our current study now demonstrates that selective activation of PPG neurons results in a complex tri-phasic response in MT/Cs. This tri-phasic response is uniquely characterized by a brief activation, which establishes a transient activation of GC feedforward inhibition, followed by a rebound long-lasting excitation (Fig. 6b). In MCs not displaying spontaneous activity and receiving more inhibitory inputs, PPG neuron activation was not sufficient to depolarize the M/TCs and resulted solely in a reinforcement of lateral inhibition. Taking together, these data suggest that PPG neurons serve to increase the contrast of the pattern of activation of olfactory output, by increasing the response of the cells with high activity and increasing the inhibition of low spiking M/TCs. Alternatively, in neurons with prominent spontaneous activity, the multiphasic inhibition-rebound excitation may serve the role of temporal control of M/TC spike patterns and in shaping the spike output. Recent work has demonstrated the importance of M/TC-selective lateral inhibition in the representation of odors and the timing of spike generation^38,40,41^. In MCs, lateral inhibition is thought to be mainly mediated at the GL level by periglomerular cells in comparison to the inhibitory inputs generated at the EPL level by GCs^42^; however, PPG neurons do not promote long-lasting inhibition but rather a brief window of inhibition/excitation allowing a reset of spiking synchronicity of the M/TCs.

At their resting potential, PPG neurons do not show any spontaneous firing activity, and a low rate of sEPSCs, and sIPSCs, hence we can hypothesize that they receive centrifugal projections from the central nervous system. Feedback from the piriform cortex has been shown to regulate global inhibition in the olfactory bulb^27,43^. Boyd and collaborators demonstrated direct excitatory projections from the pyramidal cells in the piriform cortex to the dSACs in the olfactory bulb. Our findings demonstrate a new class of glutamatergic neurons in the GCL of the olfactory bulb. *In vitro*, optical activation of molecularly-identified, PPG neurons results in a multiphasic response in the M/TCs. This type of time locking response ensures the ability for the PPG neurons to increase the contrast between spiking and silent M/TCs. This important function of the PPG – M/TC – GC microcircuit may refine olfactory perception or allow neuromodulation of the contrast during different nutritional states given the potential co-release of the GLP-1 hormone.

## Materials and Methods

### Ethical approval

All experiments described in this report were approved by the Florida State University Institutional Animal Care and Use Committee (IACUC) under protocol #1733, and were conducted in accordance with the American Veterinary Medicine Association (AVMA), the National Institutes of Health (NIH), and the UK Home Office Regulations under the Scientific Procedures Act 1986. In preparation for olfactory bulb slice electrophysiology, mice were anesthetized with isoflurane (Aerrane; Baxter, Deerfield, IL, USA) using the IACUC-approved drop method and then were killed by decapitation (AVMA Guidelines on Euthanasia, June 2007).

### Mouse lines and animal care

Detection of preproglucagon (PPG) neurons expressing a red fluorescent protein (RFP) was achieved by crossing Rosa26-tandem-dimer red fluorescent protein (tdRFP) reporter mice (Gt(ROSA)26Sor^tm1Hjf^)^11^ with mice expressing Cre recombinase under the control of the preproglucagon promoter (GLU-Cre12 mice, Tg(Gcg-icre)12Fmgb)^12^. For simplification, homozygous progeny resulting of the breeding of GLU-Cre12 and Rosa26 tdRFP mice will be referred as PPG-Cre-RFP in this manuscript. Briefly, GLU-Cre12 mice were created using a construct based on the bacterial artificial chromosome (BAC) RP23–343C17 (Children’s Hospital Oakland Research Institute, Oakland, CA, USA) in which the sequence between the proglucagon start codon in exon 2 and the stop codon in exon 6 was replaced by iCre using Red/ET recombination technology^44^ (Genebridges, Heidelberg, Germany). PPG-Cre-RFP homozygous offspring were back-crossed into C57BL/6 mice for at least 7 generations. Channelrhodopsin-2 (ChR2) was expressed in PPG neurons by crossing to heterozygosity PPG-Cre-RFP mice with the Ai32 line (B6;129S-Gt(ROSA)26Sor^tm32(CAG-COP4*H134R/EYFP)Hze^/J, Stock# 012569, RRID:IMSR_JAX:012569, Jackson Laboratories, Bar Harbor, ME, USA) that contains a floxed allele expressing the fusion protein ChR2(H134R)-EYFP in the presence of the CRE recombinase in PPG neurons^18^. Heterozygote Swiss Webster GAD67-EGFP knock-in mice (CB6-Tg(Gad1-EGFP)G42Zjh/J, RRID:IMSR_JAX:007677)^45^ were a generous gift from Dr. Pradeep Bhide (College of Medicine, The Florida State University) and were bred to PPG-Cre-RPF mice to analyze GAD67 expression in the PPG neurons in the GCL. Given that GAD67 mice are kept as heterozygotes, we found an expression of the GFP only in approximately half of the mice resulting from the crossing (4/9 mice).

All mice were housed at the Florida State University *vivarium* in accordance with the institutional requirements for animal care. All mice used in this study (*Mus musculus*, C57BL/6, 129 S, and Swiss Webster background strains) were maintained on a standard 12 h/12 h light/dark cycle and were allowed *ad libitum* access to 5001 Purina Chow (Purina, Richmond, VA, USA) and water. Mice were not treated with different conditions or derived of different experimental genotypes so no blinding was performed to the investigator in assigning mice to studies for electrophysiology or immunocytochemistry. A total number of 55 crosses were established to generate mice for our combined experiments and progeny from these breedings were randomly assigned to a given experiment. Mice were never used for multiple experiments due to different preparation needs between electrophysiology and immunocytochemistry. Mice were of both sexes and ranged from postnatal day 21 to 35 for electrophysiology and 1–2 months for immunocytochemistry. Because our vivarium is set for reverse light phase, mice were sacrificed for experiments between 3–5 hours (h) into the dark phase.

A total of 144 mice were used in our study. A total of 15 mice were excluded from the study that did not have strong ChR2 expression in the PPG-Cre-RFP x Ai32 progeny. A total of 16 mice were excluded from histological analyses that were used to optimize antigenicity in piloting the project.

### Solutions, reagents, and antisera

Phosphate-buffered saline (PBS) was made as described previously^46^. Blocking solution contained: 3% bovine serum albumin (BSA)/PBS and 1% triton X-100. All intracellular pipette solutions were used as specified in the results section. Cesium-methanesulfonate-based intracellular solution (CsMeSO_3_) used in voltage-clamp experiments contained (in mM): 120 cesium methanesulfonate, 20 tetraethylammonium chloride, 5 4-aminopyridine, 1 ethylene glycol-bis(2-aminoethylether)-N,N,N′,N′-tetraacetic acid (EGTA), 10 HEPES, 10 MgCl_2_, 0.4 NaGTP, and 2 NaATP (pH 7.3; 280–285 mOsm). Potassium-gluconate-based solution (KGluc) used in some voltage-clamp and the majority of current-clamp experiments, as noted, contained (in mM): 135 potassium gluconate, 10 KCl, 10 HEPES, 10 MgCl_2_, 0.4 NaGTP, and 2 NaATP (pH 7.3; 280–285 mOsm). Potassium-methanesulfonate-based solution (KMeSO3) used in some current-clamp experiments contained (in mM): 140 KMeSO_3_, 1.1 EGTA, 10 HEPES, 1 MgCl_2_, 0.4 NaGTP, and 2 NaATP (pH 7.3; 280–285 mOsm). Artificial cerebral spinal fluid (ACSF) contained (in mM): 119 NaCl, 26.2 NaHCO_3_, 1 NaH_2_PO_4_, 2.5 KCl, 1.3 MgCl_2_, 2.5 CaCl_2,_ and 22 D-glucose (pH 7.3; 310–315 mOsm). Sucrose-modified ACSF was used for sectioning and contained (in mM): 83 NaCl, 26.2 NaHCO_3_, 1 NaH_2_PO_4_, 3.3 MgCl_2_, 0.5 CaCl_2_, 72 sucrose, and 22 D-glucose (pH 7.3; 315 mOsm)^47^. All salts and sugars were purchased from Sigma-Aldrich (St. Louis, MO, USA) or Fisher Scientific (Pittsburgh, PA, USA).

Tetrodotoxin (TTX, voltage-gated sodium channels blocker), gabazine, AP_V_ ((2 R)-amino-5-phosphonovaleric acid, NMDA receptor antagonist), and NBQX (2,3-dihydroxy-6-nitro-7-sulfamoyl-benzo[f]quinoxaline-2,3-dione, AMPA receptor antagonist) were purchased from Abcam Biochemicals (Cambridge, MA, USA). 4-(N-ethyl-N-phenylamino)−1,2 dimethyl-6-(methylamino) pyrimidinium chloride (ZD-7288) and 4-aminopyridine (4-AP) were purchased from Sigma-Aldrich.

GAD65 was detected using a mouse monoclonal antibody, clone GAD-6, recognizing an epitope localized on the C-terminus of the protein (1:1000, cat# ab26113, RRID:AB_448989, Abcam)^37^. The GAD67 protein was detected using mouse monoclonal antibody, clone 1G10.2 (1:1000, cat# MAB5406, RRID:AB_2278725, Millipore, Billerica, MA, USA)^48^. Rat anti-muscarinic acetylcholine receptor 1α monoclonal antibody was used to detect the presence of the receptor in PPG neurons (1:1000, Millipore). Vesicular GABA transporter (VGAT) was labelled using a rabbit polyclonal serum (1:1000, BOSTER Biological Technology Pleasanton, USA). Kv2.1 protein was detected using a mouse monoclonal antiobody (Clone K89/34) obtained from UC Davis/NIH Neuromab facility (1:1000, Davis, CA).

In some cases, the red fluorescence from the Rosa26tdRFP in PPG neurons was amplified using a rabbit DsRed polyclonal antibody (1:1000, cat#632496, RRID:AB_10013483, Clontech Laboratories Inc., Mountain View, CA, USA). Host-specific Cy3, Cy2, or Alexa 647 secondary antisera were purchased from Jackson ImmunoResearch (West Grove, PA, USA), used per manufacturer’s suggested protocols, and applied at a dilution of 1:400 in blocking solution.

### Surgeries

To comprehensively examine the phenotype of the GLU-Cre-RFP mice, injections of Cre-dependent adeno-associated virus were made into the GCL (relative to bregma, 0.45, ± 0.75, 0.23 mm). Mice were initially anesthetized in an induction chamber primed with isoflurane and subsequently mounted to the stereotaxic apparatus (Stoelting Co., IL) where they were maintained under anesthetic state [2% isoflurane gas mixed with oxygen (0.5 L/min)]. AAV5 containing pAAV-EF1a-double floxed-hChR2(H134R)-EYFP-WPRE-HGHpA 1 × 10¹³ vg/mL (Addgene, Watertown, MA) were delivered bilaterally using glass 10 µL Hamilton syringes (O.D. ~15–20 μm) (1.5 µL per olfactory bulb) at a rate of 100 nL/min. The analgesic buprenorphine was administered immediately following surgery and at 12 h intervals for one day following surgery (0.05 mg/kg). Mice were sacrificed 15 days post-surgery for anatomical analyses.

### Olfactory bulb slice electrophysiology

A total of 47 mice were used for 165 patch-clamp electrophysiological recordings from olfactory bulb slices. Following isoflurane anesthesia, mice were killed by decapitation (see Ethics Approval). Olfactory bulbs were dissected and coronal sections (275–300 μm) were prepared as previously described^4,47^. The blue light source (Polygon 400, Mightex, Pleasanton, CA, USA) used to stimulate channel rhodopsin at 470 nm was mounted on the side of the microscope to a dichroic mirror located between the binoculars and the objective, allowing the light to be directed toward the slice through the 40 × Zeiss objective. The spatial illumination pattern on the slice was controlled by Dynamic Spatial Illuminator software (Mightex, version 2.0.0) in conjunction with a Zeiss Axiocam digital camera and associated AxioVision software (version 4.8, Carl Zeiss Microimaging, RRID:SCR_002677). The duration and intensity of the light stimuli were set in BioLED source control module software (Mightex, version 1.0.2). The light stimulus was controlled in pClamp 10.5 software (Axon Instruments/Molecular Devices, Sunnyvale, CA, USA) using a digital output channel to connect the digitizer (Digidata 1440 A, Axon Instruments/Molecular Devices) to the BioLED controller.

Membrane voltage and current recordings were generated using pClamp 10.5 software in conjunction with an Axopatch 200B amplifier (Axon Instruments/Molecular Devices, RRID:SCR_011323). The analog signal was filtered at 2 kHz and minimally digitally sampled every 50 μs (20 kHz). Electrodes were fabricated from borosilicate glass (Hilgenberg no. 1405002, Malsfeld, Germany) to a diameter of approximately 2 μm to yield pipette resistances ranging from 4 to 7 MΩ. Positive pressure was retained while navigating through the olfactory bulb laminae until a high resistance seal (Re = 2–10 GΩ) was obtained on a cell in the slice^4,49^. The morphology and biophysical properties of the neurons were used to distinguish M/TCs from GCs^1^. All neurons within the MC layer were designed as M/TCs and were not distinguished from tufted cells. Neurons within the GCL were classified as either GCs or PPG neurons. PPG neurons were identified by excitation of the RFP. We previously biophysically characterized these neurons (Thiebaud *et al*., 2016) as a subclass of neurons in the GCL called deep short-axon cells (dSACs), also named Cajal cells, Blanes cells, or Golgi cells, as previously described by Eyre and collaborators^1–3,9,10^. Cell-attached recordings were made when the resistance was above 1 GΩ with pipettes filled with intracellular solution. The whole-cell configuration was established by applying gentle suction to the lumen of the pipette while monitoring resistance. The pipette capacitance was electrically compensated through the capacitance neutralization circuit of the Axopatch 200B amplifier. Likewise, series resistance compensation was used to electrically reduce the effect of pipette resistance.

Each neuron was sampled for adequate resting potential (less than –55 mV) and proper series resistance (less than 40 MΩ) right after rupturing the patch prior to initiating a series of current-clamp recordings. Voltage-clamp traces were subtracted linearly for leakage conductance. Resting membrane potentials were corrected for a –14 mV junction potential offset. In voltage-clamp, excitatory postsynaptic currents (EPSCs, V_h_ = –70 mV) and inhibitory postsynaptic currents (IPSCs, V_h_ = 0 mV) were recorded in multiple epochs of 1–5 s. The inter-stimulus interval was always set to a minimum of 20–30 s to allow for a full recovery of the activated channels.

### Immunofluorescence

Mice were intracardially perfused with 4% paraformaldehyde in PBS (PFA/PBS). The OBs were post-fixed overnight in 4% PFA/PBS, decalcified for 3–5 days in 0.3 M ethylenediaminetetraacetic acid (EDTA), and then prepared for cryosectioning (12 or 20 µm) as previously described in Thiebaud *et al*., 2014^50^. Prepared brain frozen sections were air-dried for 30 minutes (min), washed 3 times in PBS, and then incubated for 30 min in blocking solution to prevent non-specific binding. After the blocking step, the tissue sections were incubated overnight at 4 °C with the primary antiserum diluted in blocking solution. The immunofluorescence signal was detected after a 2 hour (h) incubation at room temperature (rt) with host-specific secondary antisera. Sections were stained with 4,6-diamidino-2-phenylindole (DAPI) nuclear stain diluted in PBS (1:15,000, Life Technologies, Carlsbad, CA) and coverslipped with Fluoromount G (Southern Biotechnology, Birmingham, AL, USA) to prevent photobleaching. Olfactory bulb sections were examined by fluorescence using either a Zeiss inverted microscope (Axiovert S100) or a Zeiss confocal microscope (LSM 880). Digital images were captured with a Zeiss Axiocam digital camera and associated AxioVision (version 4.8, Carl Zeiss Microimaging, RRID:SCR_002677) or Zen lite (version 2.1, RRID:SCR_013672) software.

### Experimental design and and statistical analyses

All electrophysiological data were analyzed using Clampfit 10.5 (Axon Instruments/ Molecular Devices, RRID:SCR_011323), Origin 8 (OriginLab, Northampton, MA, USA, RRID:SCR_002815), and Igor Pro 6.12 A (WaveMetrics Inc., Portland, OR, RRID:SCR_000325) software with the NeuroMatic version 2 plug-in (written by Jason Rothman, RRID:SCR_004186). Rise and decay time constants of postsynaptic currents were analyzed starting from the middle of the light pulse between 10 and 90% of the event maximal amplitude. All electrophysiological data are available by contacting the D. Fadool Laboratory and are supplied in pClamp v10 acquisition compatible files (*.abf).

For statistical analyses, Prism 6.0 software (GraphPad Software, Inc., La Jolla, CA, USA, RRID:SCR_002798) was used to perform the Friedman test (non-parametric alternative for repeated-measures analysis of variance; ANOVA) followed by Dunn’s multiple comparison *post*-*hoc* test (Fig. 3d’,g’), the Wilcoxon signed-rank test (Figs 3e’,f’,4d’), or the Student’s *t*-test for 2-sample comparison (Table 1). All data are reported as the mean ± standard deviation. Different letters denote statistically-different mean values with a corresponding *p* value as specified. Statistical difference was defined at the 95% confidence interval, or α ≤ 0.05. Power analyses for each experiment were calculated using G*Power (Version 3.1.9.2, Franz Faul, Kiel University, Germany, RRID:SCR_013726). A minimum power (1-β) of 80% was considered acceptable for the experiments. The sample size tested for a range of estimated means and SD was considered adequate with a power (1-β) superior to 0.80, with α ≤ 0.05. Computed powers (1-β) were the following: Fig. 2f: 1-β = 1.0; Fig. 3d’: 1-β = 0.96; Fig. 3e’: 1-β = 1.0; Fig. 3f’: 1-β = 1.0; Fig. 3g’: 1-β = 0.82; Fig. 4d’: 1-β = 0.87.

## Data Availability

All data generated or analyzed during this study are included in this published article. Transgenic mice lines were generated by professors Gribble, and Reimann and can be made available through material transfer agreements (MTA) arranged with these authors. The communicating author is happy to arrange shipment of mice to individuals in the USA following completion of the MTA while other co-authors will arrange shipment to other locations.
